# Development of a 3D-Printed Capacitive Sensor for Soil Water Content Estimation Using Nickel-Based Conductive Paint

**DOI:** 10.3390/s26051494

**Published:** 2026-02-27

**Authors:** Alessandro Comegna, Shawkat B. M. Hassan, Antonio Coppola

**Affiliations:** 1 Department of Agricultural Forestry Food and Environmental Sciences (DAFE), University of Basilicata, 85100 Potenza, Italy; shawkat.hassan@unibas.it; 2 Department of Chemical and Geological Sciences, University of Cagliari, 09042 Cagliari, Italy; antonio.coppola@unica.it

**Keywords:** capacitive-based sensors, low-cost systems, 3D printing, conductive paint, soil sensors, soil water content

## Abstract

**Highlights:**

**What are the main findings?**

- Development of a low-cost capacitive sensor.
- Consistent and reliable performance.

**What are the implications of the main findings?**

- It is possible to build the device on one’s own.
- The device is suitable for monitoring soil water content with acceptable accuracy.

**Abstract:**

Understanding hydrological, agricultural, and environmental processes in soils relies on accurately measuring volumetric water content (θ), matric potential (*h*), and hydraulic conductivity (*K*). These parameters are fundamental for quantifying plant-available water, optimizing irrigation scheduling in precision agriculture, modeling watershed responses, and studying the impacts of climate change in complex ecosystems. Among these parameters, θ is truly indispensable, as it represents the primary indicator of the water status of soils and a prerequisite for interpreting the other hydraulic variables. In recent years, capacitive sensors have become one of the most widely adopted technologies for θ estimation, owing to their favorable balance between accuracy, robustness, and affordability. These sensors infer soil moisture by measuring dielectric permittivity of soils, which is strongly governed by water content, making them particularly suitable for distributed monitoring and IoT-based environmental applications. The present study aimed to develop a low-cost capacitive sensor for θ estimation. This sensor can be made using 3D printing technology combined with conductive, nickel-based paint, which (once applied on the 3D-printed guides) forms the capacitive electrode. The capacitive component operates at an operational frequency of 60 MHz. The system was subjected to a rigorous testing protocol, including calibration and validation phases under laboratory conditions using three soils of different textures. Its performance was specifically compared with the time-domain reflectometry (TDR) technique, which is widely recognized in Soil Physics and Soil Hydrology as the reference method for θ estimation due to its reliability and accuracy. These tests confirmed the effective performance of the proposed sensor, which overall exhibited good reliability within the selected validation range, corresponding to a θ range of 0 to 0.40 cm^3^/cm^3^.

## 1. Introduction

The volumetric water content (i.e., volume of water in the soil/total volume of the soil) represents one of the most informative variables for describing the physical state of soils. Its measurement is crucial for interpreting how water is stored, transported, and made available to plants, and it plays a central role in applications ranging from irrigation management to the evaluation of environmental impacts. Without reliable θ data, the understanding of key hydrological processes remains incomplete [1,2,3,4,5,6,7,8].

The measurement of θ has evolved significantly in the last few decades, with a prominent shift from direct, destructive methods (e.g., gravimetric sampling) towards indirect, automated sensing techniques. Among such techniques, capacitive sensors have gained widespread adoption, especially in recent years [9,10,11,12,13]. These sensors operate by measuring the soil’s dielectric permittivity, which is predominantly influenced by water content, to estimate θ [14,15,16]. Their popularity stems from a favorable balance between cost, robustness, and accuracy, making them suitable for integration into distributed sensor networks [17,18,19]. Recently, the drive for extensive spatial monitoring has catalyzed a low-cost approach in sensor development [20,21,22]. This paradigm focuses on leveraging open-source hardware, simplified electronics, and Internet of Things (IoT) communication protocols to create affordable, yet reliable, monitoring systems [23,24]. While these low-cost capacitive sensors may initially present challenges related to calibration and soil-specific sensitivity, ongoing technological refinements such as operating at higher frequencies to mitigate salinity effects are steadily enhancing their performance [25,26,27,28]. Various studies have also indicated that these effects are substantially minimized when capacitive sensors operate in soils with temperatures of 15–30 °C and soil solution EC_w_ under roughly 10 dS/m [13,15,29,30,31,32]. Nevertheless, capacitive sensors still require proper calibration and careful management to maintain measurement accuracy under variable environmental conditions and to ensure long-term system reliability, particularly in remote or harsh settings [2,33,34,35,36].

In the present paper the aim of our research was to design and implement a compact capacitive-based sensor for soil water content measurements. The sensor, namely MoCap60 (Moisture Capacitive 60 MHz), offers an innovative low-cost solution for real-time monitoring of θ, delivering reliable and accurate measurements. The sensor integrates low-power wireless technology designed for extended field operation, providing a cost-effective measurement system suitable for large-scale environmental monitoring. To avoid classical measurement problems, we chose an operational frequency of 60 MHz, which is particularly suitable for measurements in soils, as in the range of approximately 50–100 MHz, the effects of temperature and salinity are significantly reduced [37]. Furthermore, at this frequency, the dielectric dispersion effect, which may affect soils containing moderate to high amounts of clay minerals, is also less pronounced [38,39,40].

The MoCap60 sensor can be easily assembled, as its structural components are produced through 3D printing technology. Two plastic guides, once coated with a nickel-based conductive paint, serve as capacitive electrodes. This paper presents the design and implementation of the proposed MoCap60 sensor and provides detailed information on the series of laboratory-controlled tests carried out to calibrate and validate the device using three soils with different textures.

## 2. Materials and Methods

### 2.1. Hardware Description

The MoCap60 sensor consists of: (i) an ESP32 mini microcontroller board (Espressif, Shanghai, China); (ii) a pair of 3D-printed plastic guides coated with nickel-based conductive paint (841AR–Super Shield™ Nickel Conductive Coating, MG chemicals, Burlington, ON, Canada), which form the electrodes for soil moisture estimation; (iii) a waterproof DS18B20 temperature sensor (Dallas Semiconductor, Dallas, TX, USA) used as a reference; and (iv) a microSD module for data storage.

**Figure 1:**
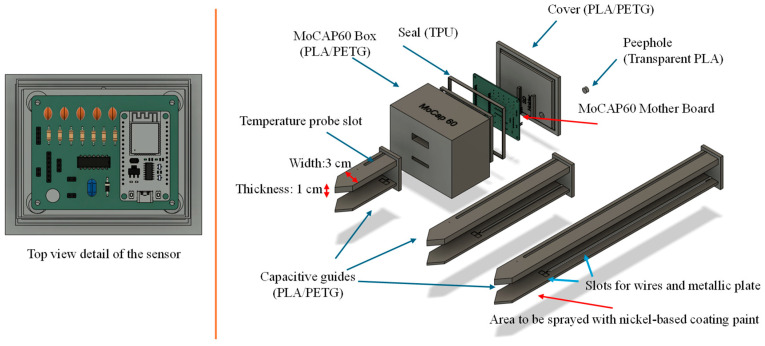
Illustration of the MoCAP60 sensor, showing its main components and the recommended materials for their fabrication. The figure also highlights the nickel-coated capacitive guides, and the dedicated slot designed to house the DS18B20 temperature probe.

Figure 1 shows the sensor as it appears after being manufactured with a 3D printer.

The accessory capacitive guides (for measurements at –5 cm, –15 cm, and –30 cm below the soil surface) are also displayed. These guides (3 cm wide and 1 cm thick) are designed to also hold the temperature sensor, which is required since capacitive sensing is known to be temperature-dependent.

The capacitive electrodes can be positioned at different depths, and both guides must be coated with the conductive paint. Before applying the paint, the capacitive system must be wired using two leads (model type AWG30), each soldered to a stainless steel plate of specific dimensions (6 mm × 7 mm), which are then inserted into the dedicated slots built into the guides. The electronic components are placed inside a waterproof box while a TPU seal ensures complete water-proofing of the sensor.

Preliminary tests were performed to verify that the coating thickness and application method did not introduce measurable alterations in sensor response. The paint layer was applied uniformly and kept as thin as possible to avoid affecting the sensor geometry and electrical field distribution. Within the tested conditions, no significant differences in sensor output were observed.

As introduced earlier, the MoCAP60 sensor’s capacitive module operates with a 60 MHz oscillator. This frequency is generated by a Pierce-type oscillator circuit integrated into the sensor, which uses a quartz crystal. In addition to the crystal, the circuit incorporates resistors, capacitors, and inductors, together with a digital inverter, allowing the crystal to oscillate at the third overtone frequency, ensuring stable and precise operation [41].

The MoCAP60 sensor measures changes in the dielectric properties of the soil, which are directly related to the θ values. When the water content in the soil changes, the dielectric constant of the soil changes accordingly, which alters the capacitance between the sensor electrodes. This capacitance variation is converted into a voltage output (*V_out_*) by the sensor’s internal circuitry.

The general relationship between the volumetric water content θ and $V_{out}$ can be expressed as:

(1) $$\theta =a\cdot \left(1/V_{out}\right)+b$$

where a and b are soil-dependent calibration coefficients. Equation (1) can be modified by also accounting for the temperature factor as follows:

(2) $$\theta_{T_{ref}}=a\cdot \left(1/V_{out}\right)+b+c\cdot \left(1/V_{out}\right)\left(T-T_{ref}\right)$$

where c is the temperature compensation coefficient, and $T_{ref}$ is the reference temperature (e.g., 25 °C).

**Figure 2:**
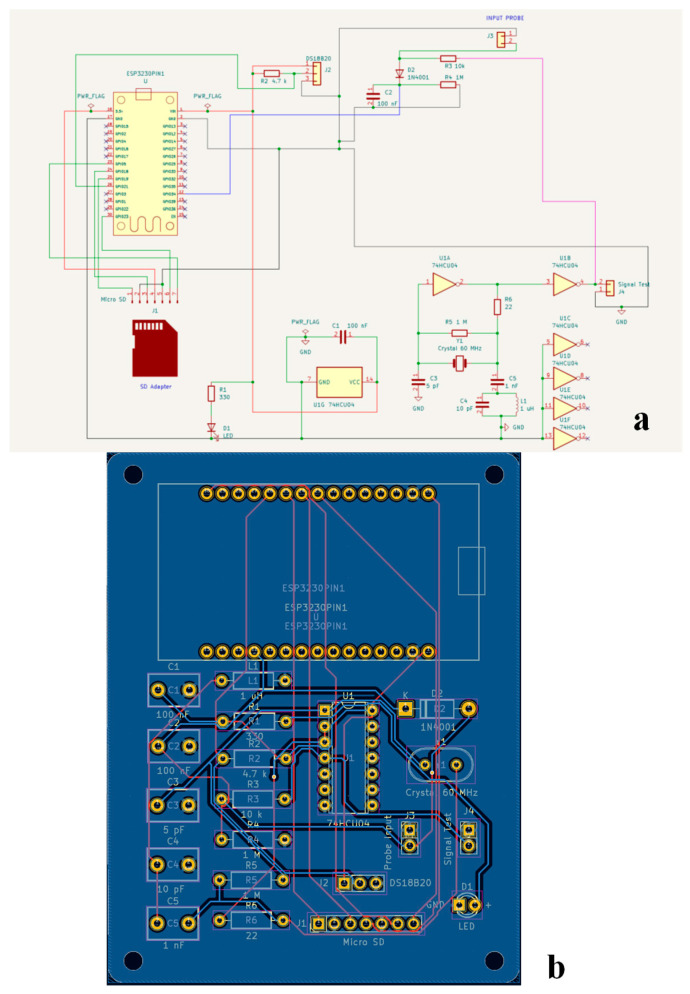
(**a**) Electric circuit diagram of MoCAP60, and (**b**) two-layer printed circuit board (PCB) generated by Kicad software.

Figure 2a,b present the electrical wiring layout and the two-layer printed circuit board (PCB) of the MoCAP60 sensor, which was designed and implemented using the KiCad software platform (vers 8.0, https://www.kicad.org/).

A detailed list of the components used to assemble the sensor, also known as the Bill of Materials (BOM), is provided in Table 1. Detailed information on the MoCAP60 sensor, including the source code (see file MoCAP60.ino) controlling its hardware, is provided in the Supplementary Material accompanying this paper.

**Figure 3:**
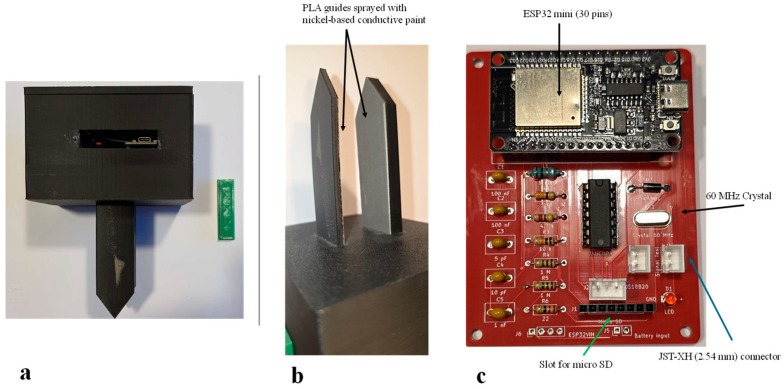
MoCAP60 hardware: (**a**,**b**) main components; (**c**) details of the two-layer printed circuit board (PCB) assembled with all the electronic parts.

The device is also equipped with an external microSD card slot for storing measurements (see Appendix A for further information on this aspect). The MoCAP60 device can be powered either by a Li-ion battery (e.g., 3.7 V, 1000 mAh) or via a USB-C cable. Figure 3a–c show the main components of the sensor and the two-layer PCB, fully assembled with the electronic components previously listed in Table 1.

Comprehensive documentation, including the firmware managing MOCAP60′s, hardware functionalities, and details on production costs, is available in Appendix B.

### 2.2. Soil Properties and Experimental Setup

To assess the calibration, validation, and performance of the MoCAP60 sensor, three sets of experiments (labeled as exp#1, exp#2, and exp#3) were carried out. The laboratory tests were conducted on repacked samples prepared from three soils with different textures. Based on the IUSS Working Group WRB (2006) classification [42], the soils corresponded to sand (SAND), sandy loam (SALO), and silty loam (SILO). Their main physical and chemical characteristics are reported in Table 2.

Soil texture was assessed using the method proposed by [43], while bulk density (*ρ_b_*) was obtained following the approach of [44]. The *pH* and organic carbon (*OC*) contents were quantified using the analytical procedures reported by [45,46]. The electrical conductivity of the water used throughout the experiments was 0.17 dS m^−1^, as measured with a Cyberscan 500 conductivity meter.

**Figure 4:**
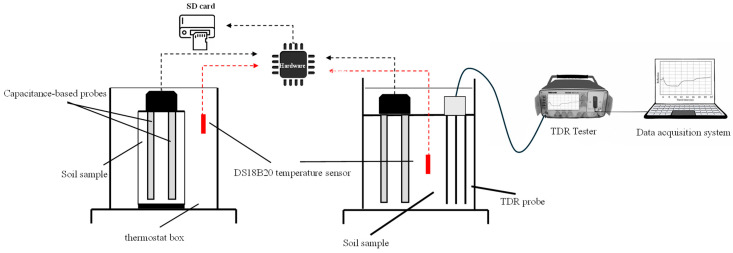
Experimental setup used in: exp#1 (**left**) and exp#2 (**right**).

Exp#1 and exp#2 focused on the calibration and validation of the MoCAP60 capacitive sensor. For these experiments, following a procedure similar to that proposed by [1], the soil samples were oven-dried at 105 °C and sieved at 2 mm. For soil preparation, known amounts of soil and water were mixed together, and then kept for 12 h in sealed plastic bags to prevent any evaporation. Finally, soil was repacked in PVC cylinders (11 cm in height and 20 cm in diameter). Figure 4 illustrates the experimental setup used for these tests.

After preparing the soil samples, the capacitive probes used for θ determination were placed vertically within the soil sample. To minimize water loss throughout the tests, both ends of the PVC cylinders were tightly sealed with plastic film. The experimental procedure involved a comprehensive set of measurements aimed at characterizing the sensor response over a broad moisture range in all three soils, covering θ values from 0 to 0.40 cm^3^/cm^3^. Readings were collected at discrete moisture levels of 0, 0.10, 0.15, 0.20, 0.30, and 0.40.

Considering that capacitive measurements are influenced by temperature [29,47], each test (corresponding to a specific θ level) was carried out while gradually varying the temperature of the soil sample from 20 °C to 30 °C (which is the modal temperature range in many temperate climates) in 1 °C increments inside a thermostatic chamber. During the experiments, the sensor output voltage (*V_out_*) was continuously recorded. At the same time, the actual volumetric water content was independently quantified using the thermo-gravimetric procedure described by [48]. These datasets were subsequently used to establish the calibration curve linking θ to the measured voltage.

For the MoCAP60 validation (exp#2), a new dataset was prepared for each soil, following the same protocol as used during the calibration phase (i.e., exp#1). In this stage measurements were also taken using the well-proven technology of time domain reflectometry (TDR), exhibiting sufficient sensitivity for characterization of volumetric water content in soils [26,49,50,51,52,53,54,55]. For TDR measurements the Tektronix model 1502C (Tektronix Inc., Baverton, OR, USA) was used, connected to a three-wire probe, with 10 cm long waveguides connected to the tester by a 2-m-long coaxial cable. For these experiments, the MoCAP60 sensor and TDR probes were placed in a soil sample 400 mm high and 300 mm in diameter.

**Figure 5:**
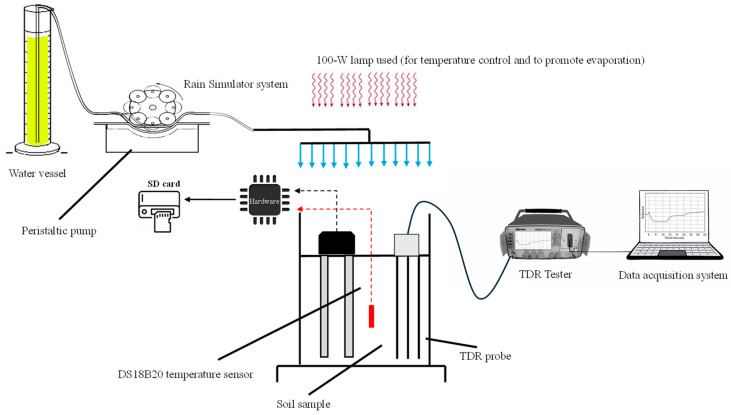
Experimental setup used in exp#3.

Finally, after calibrating and validating the capacitive module of the sensor, a final test (exp#3) was carried out, with reference to SALO soil, to assess the overall performance of the MoCAP60 device. In exp#3, a drying–wetting cycle with controlled rainfall was performed inside a thermostat box at a constant temperature of 25 °C. During the experiment, θ measurements were recorded over time using both the MoCAP60 sensor and a TDR device (Figure 5). For this test, the sensor probes were inserted into the same cylinder used for exp#2.

The experiment lasted a total of 72 h. Every 10 h, a rainfall event with an intensity of 10 mm h^−1^ was applied for 30 min. The simulated rainfall was uniformly distributed over the soil surface using a rain simulator; evaporation, from the soil surface, was induced with a 100-W lamp. During the test, MoCAP60 and TDR measurements were taken respectively every 6 min and 5 h.

### 2.3. Performance Assessment of the MoCAP60 Sensor Through Statistical Indices

The performance of the MoCAP60 sensor was assessed using three statistical indicators [56,57]: (i) the mean absolute percentage error (*MAE*), (ii) the mean bias error (*MBE*), and (iii) the model efficiency (*EF*):

(3) $$MAE\left(\%\right)=\frac{\left|E_{i}-O_{i}\right|}{N}\cdot 100$$

(4) $$MBE=\frac{\sum_{i=1}^{N}\left(E_{i}-O_{i}\right)}{N}$$

(5) $$EF=1-\frac{\sum_{i=1}^{N}{\left(E_{i}-O_{i}\right)}^{2}}{\sum_{i=1}^{N}{\left(O_{i}-\bar{O}\right)}^{2}}$$

where ***O_i_*** is the observed value (i.e., obtained via the thermo-gravimetric method), ***E_i_*** is the prediction or estimated (i.e., via MoCAP60 readings), $\bar{O}$ is the mean of the observed data, and ***N*** is the number of observations.

## 3. Results and Discussion

### 3.1. Sensor Calibration

**Figure 6:**
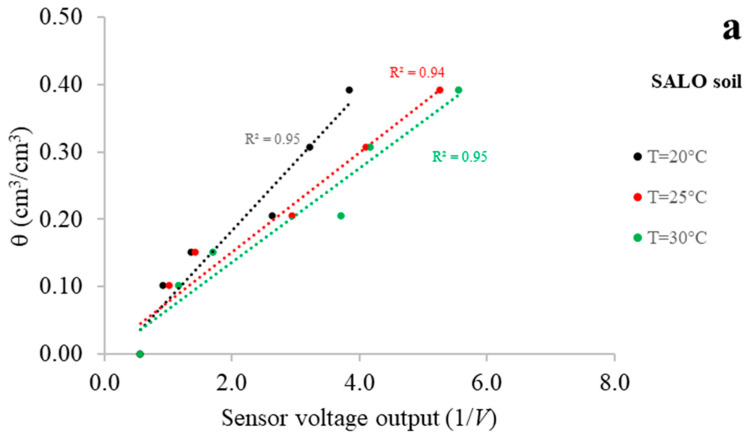

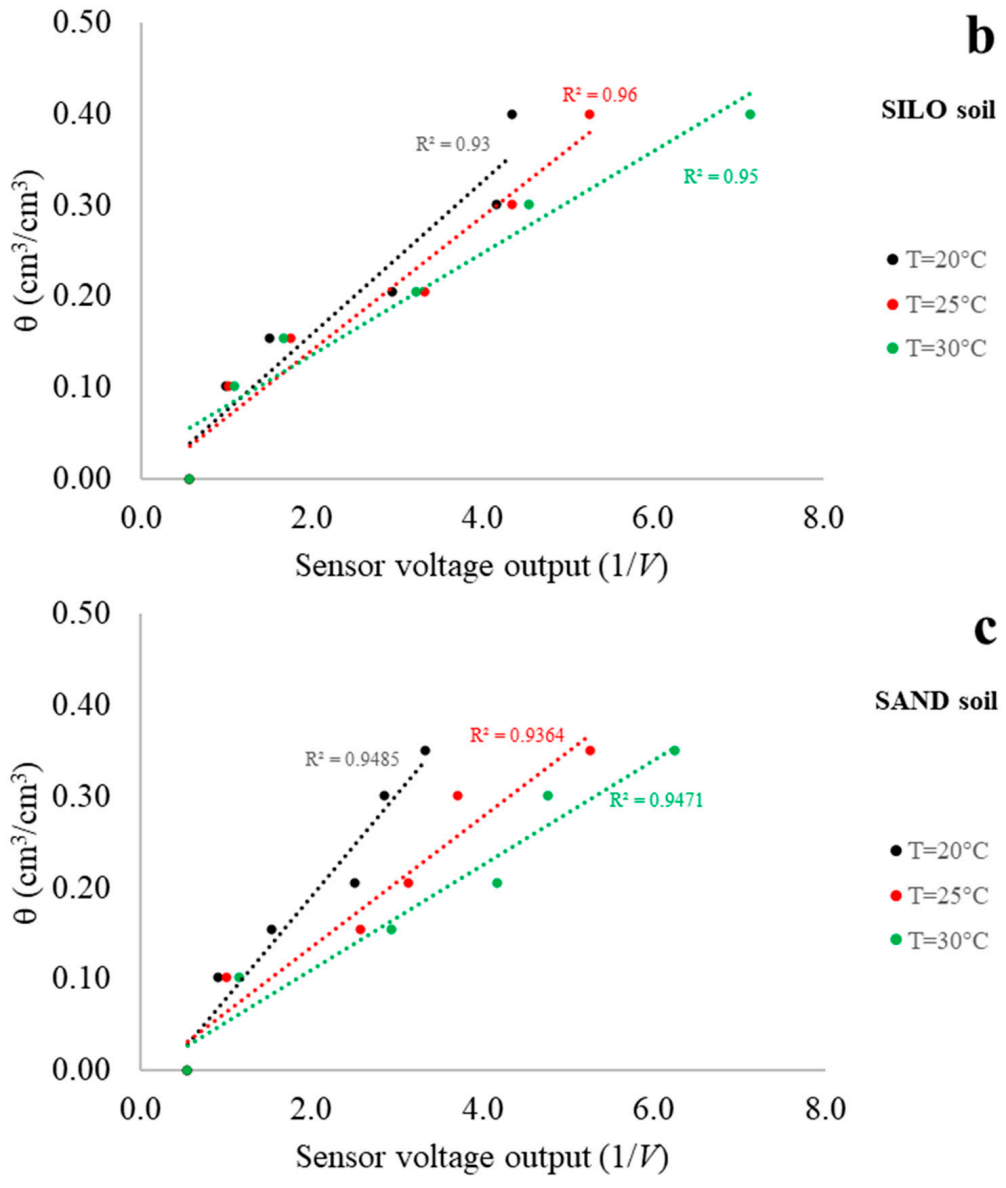
Relationship between θ values and the 1/*V* readings for SALO, SILO, and SAND soils, at three selected soil temperatures (20 °C, 25 °C, and 30 °C).

With reference to the SALO, SILO and SAND soils, the MoCAP60 sensor calibration results at three selected soil temperatures of 20 °C, 25 °C, and 30 °C are shown in Figure 6.

The outcomes of exp#1 highlight the linear temperature dependence between θ values obtained via the thermo-gravimetric method and 1/*V* values determined via the MoCAP60 sensor. It should be highlighted that, at a constant 1/*V* value, θ tends to reduce as temperature rises. In addition, when the soil approaches dry conditions, the temperature-related differences in θ values become less pronounced, reflected by the convergence of the linear fits, because the sensor’s dielectric behavior is increasingly controlled by soil (i.e., solid phase) permittivity [54,58,59,60]. Within the temperature interval used for sensor validation, all soils exhibited comparable patterns. This finding is particularly significant, as it suggests that the capacitive sensor does not require soil-specific calibration for the set of soils examined.

For the sake of completeness, Table 3 presents the estimated coefficients *a* and *b*, together with the coefficient of determination (*R*^2^) of the linear calibration equations for the three soils considered across the entire temperature domain. The high *R*^2^ values (all above 0.90) confirm the strong relationship between the volumetric water content and the sensor readings.

### 3.2. Sensor Validation

**Figure 7:**
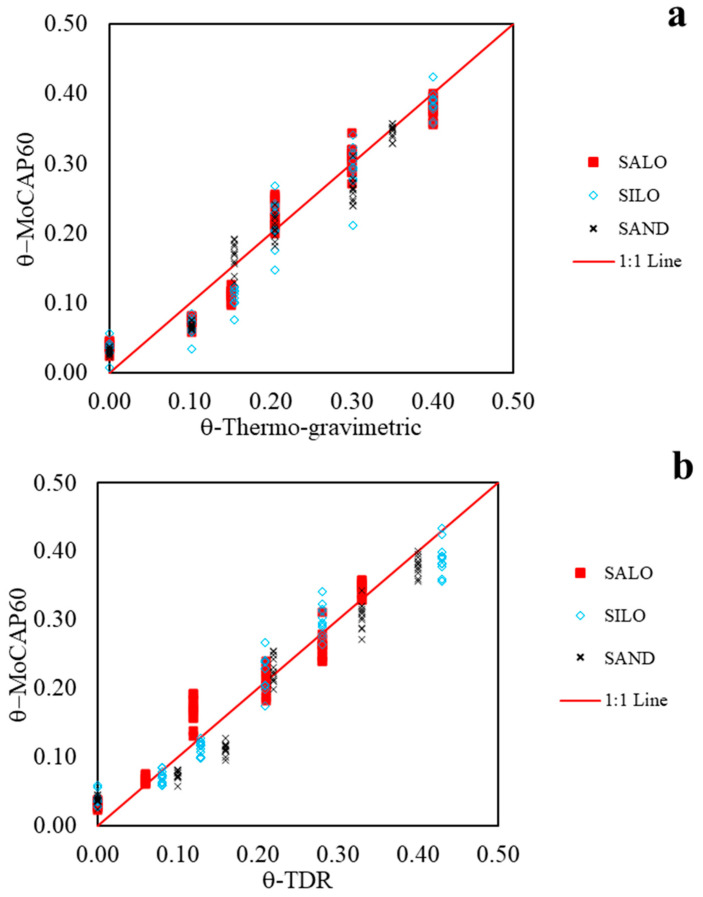
Correlation between (**a**) the θ-MoCAP60 values and θ-thermo-gravimetric values, and (**b**) the θ-MoCAP60 values vs. θ-TDR measured, in the 20–30 °C temperature range.

The validation results of the sensor are illustrated in Figure 7a, where the θ values derived from the linear calibration functions are compared with the reference θ values obtained through the thermo-gravimetric method. Figure 7b compares the MoCAP60 readings with those measured using the TDR technique. Overall, the estimated values align closely with the 1:1 line, confirming the sensor’s reliable performance.

Table 4 shows the key statistical parameters, namely *MBE*, *MAE*, and *EF*, calculated in the temperature-validation range, and across the three soil types. Part (a) of Table 4 refers to the comparison between the measurements obtained with the MoCAP60 sensor and the thermo-gravimetric method, whereas part (b) refers to the comparison between the measurements obtained with the MoCAP60 sensor and those acquired using the TDR technique.

The consistently high *EF* values, all above 0.90 for the overall performance, indicate a good predictive capability for all soil types.

Regarding the accuracy of the estimates, the *MAE* values provide a clear measure of average error magnitude. The SAND soil demonstrates the best overall precision with the lowest aggregate *MAE* (2.58), indicating its estimates are, on average, the closest to the observed values. The SALO soil follows closely, with an overall MAE of 2.7, while SILO has a slightly higher overall error (*MAE* = 2.98). This pattern is consistent at most individual depths, where SALO frequently achieves the lowest *MAE*.

An analysis of the *MBE*, which indicates systematic over- or underestimation, reveals that all models have a very small overall bias, with absolute values below 0.012. The *MBE* for SALO and SAND is negative overall (−0.0039 and −0.0059, respectively), indicating a negligible tendency to slightly underestimate θ values. SILO has the largest overall negative bias (−0.0116), suggesting a more consistent, though still small, underestimation. It is noteworthy that at 27 °C, the SAND soil type shows a positive *MBE* (0.0144), which is one of the largest single biases in the dataset.

The statistical parameters confirm that the MoCAP60 device provides a reliable response with respect to the three investigated soils. However, the SALO soil emerges as the most reliably estimated, due to its superior combination of the highest model efficiency (*EF* = 0.94), lowest overall absolute error (*MAE* = 2.72), and minimal systematic bias (*MBE* ≈ −0.004).

### 3.3. Temperature-Compensated Equations for MoCAP60 Sensor

Finally, from the experimental measurements conducted across the calibration-validation temperature range, temperature-compensated equations were derived to normalize the sensor’s output to a standard reference temperature of 25 °C. These relationships account for the combined effect of sensor output (1/*V_out_*) and soil temperature (*T*) on volumetric water content estimation.

The soil-specific calibration functions are:

- SALO soil:

(6) $$\theta_{25{}^{\circ}C}=-0.0847\cdot \left(1/V_{out}\right)-0.0177+0.0012\cdot \left(1/V_{out}\right)\cdot \left(T-25\right)$$

- SILO soil:

(7) $$\theta_{25{}^{\circ}C}=-0.0742\cdot \left(1/V_{out}\right)-0.0042+0.0006\cdot \left(1/V_{out}\right)\cdot \left(T-25\right)$$

- SAND soil:

(8) $$\theta_{25{}^{\circ}C}=-0.0670\cdot \left(1/V_{out}\right)-0.0043+0.0015\cdot \left(1/V_{out}\right)\cdot \left(T-25\right)$$

Additionally, a general calibration function valid across all three soil types was established:

(9) $$\theta_{25{}^{\circ}C}=-0.0753\cdot \left(1/V_{out}\right)-0.0087+0.0011\cdot \left(1/V_{out}\right)\cdot \left(T-25\right)$$

This general function provides a reliable estimation of volumetric water content regardless of soil type, with a coefficient of determination of *R*^2^ = 0.87. The consistency among the individual soil equations supports the validity of this generalized approach, which simplifies sensor deployment while maintaining satisfactory accuracy across different soil textures.

### 3.4. Sensor Performance Under Dynamic-Controlled Conditions

**Figure 8:**
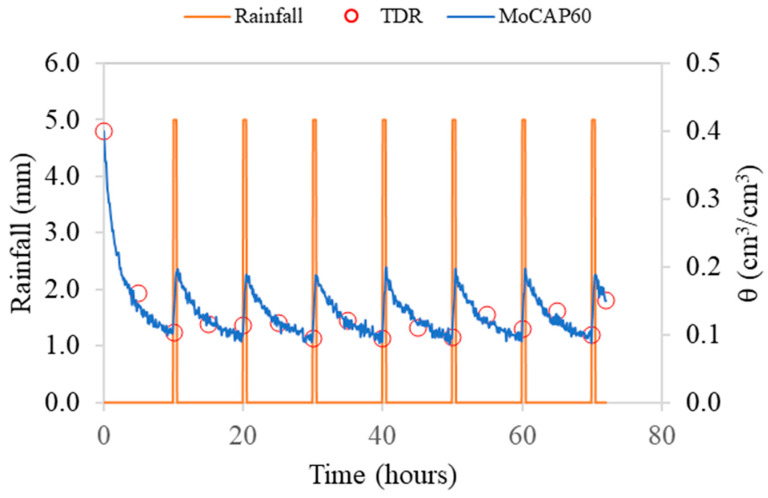
Volumetric water content measured by the MoCAP60 sensor during a controlled drying–wetting cycle. Red dots indicate TDR measurements collected every 5 h for comparison.

For exp#3, Figure 8 presents the θ measurements recorded by the MoCAP60 sensor during a drying–wetting cycle, with controlled rainfall. TDR measurements were also collected every 5 h (shown as red dots).

The figure illustrates the temporal evolution of θ measured by the MoCAP60 sensor during a controlled drying–wetting cycle, alongside the applied rainfall pulses. Each rainfall event (orange bars) produces an immediate increase in θ values, followed by a gradual decline as the soil dries. The MoCAP60 data (blue line) clearly capture these dynamics, showing consistent response patterns after each irrigation event and during the entire test. The TDR measurements (red circles), taken every 5 h, align well with the MoCAP60 readings, confirming the reliability of the sensor, throughout the entire monitoring period. Overall, the plot highlights the sensor’s ability to track soil moisture variations with high temporal resolution.

## 4. Conclusions

This study illustrates the development and validation of the MoCap60 device, a new low-cost capacitive sensor designed for real-time monitoring of soil water content. The sensor represents an effective and accessible solution, combining technical innovation with practical applicability.

The sensor’s performance was rigorously evaluated through laboratory tests on three distinct soil types. The results confirmed a high degree of reliability and accuracy, as evidenced by strong linear correlations during calibration and good statistical metrics (*MBE*, *MAE*, and *EF*) calculated during the independent validation phase. A particularly noteworthy finding was the consistent performance across different soils, which suggests that a single, generalized calibration may be sufficient for a wide range of conditions, thereby simplifying the sensor’s deployment.

Furthermore, the MoCap60 sensor effectively tracked dynamic changes in soil moisture during a simulated drying–wetting cycle, showing close agreement with measurements obtained using the TDR technique. This confirms the sensor’s capability for high-resolution monitoring in real-world scenarios. In summary, the MoCap60 sensor fulfills its design objectives, offering a robust, accurate, and affordable tool that is highly suitable for integration into large-scale networks for precision agriculture, irrigation management, and hydrological studies.

## Figures and Tables

**Table 1:** List of electronic components (BoM) required for assembling MoCAP60.

Footprint Assignment	Designator	Quantity	Footprint Specification (Kikad)	Mounting Type
C1	Capacitor:100 nF	1	C_Disc_D8.0 mm_W5.0 mm_P5.00 mm	THT *
C2	Capacitor:100 nF	1	C_Disc_D8.0 mm_W5.0 mm_P5.00 mm	THT
C3	Capacitor:100 nF	1	C_Disc_D8.0 mm_W5.0 mm_P5.00 mm	THT
C4	Capacitor:100 nF	1	C_Disc_D8.0 mm_W5.0 mm_P5.00 mm	THT
C5	Capacitor: 5 pF	1	C_Disc_D8.0 mm_W5.0 mm_P5.00 mm	THT
D1	Diode: 1N4001	1	D_DO-41_SOD81_P10.16 mm_Horizontal	THT
D2	Diode: 1N4001	1	D_DO-41_SOD81_P10.16 mm_Horizontal	THT
J1	MPX5100DP	1	PinHeader_1x03_P2.54 mm_Vertical	THT
J2	MPX5100DP	1	PinHeader_1x03_P2.54 mm_Vertical	THT
J3	Connector 01x02	1	PinHeader_1x02_P2.54 mm_Vertical	THT
J4	MicroSD	1	PinHeader_1x06_P2.54 mm_Vertical	THT
L1	Inductor: 1 μH	1	R_Axial_DIN0207_L6.3 mm_D2.5 mm_P10.16 mm_H	THT
R1	Resistor: 4k7 Ω	1	R_Axial_DIN0207_L6.3 mm_D2.5 mm_P10.16 mm_H	THT
R2	Resistor: 4k7 Ω	1	R_Axial_DIN0207_L6.3 mm_D2.5 mm_P10.16 mm_H	THT
R3	Resistor: 4k7 Ω	1	R_Axial_DIN0207_L6.3 mm_D2.5 mm_P10.16 mm_H	THT
R4	Resistor: 10 kΩ	1	R_Axial_DIN0207_L6.3 mm_D2.5 mm_P10.16 mm_H	THT
R5	Resistor: 1 MΩ	1	R_Axial_DIN0207_L6.3 mm_D2.5 mm_P10.16 mm_H	THT
R6	Resistor: 10 kΩ	1	R_Axial_DIN0207_L6.3 mm_D2.5 mm_P10.16 mm_H	THT
U1	Inverter: 74HCU04	1	Package_DIP:DIP-14_W7.62 mm	THT
U2	ESP32 mini (30 pins)	1	ESP-WROOM-32	THT
Y1	Crystal 60 MHz	1	Crystal:Resonator-2Pin_W10.0 mm_H5.0 mm	THT

* THT: Through-Hole Technology Mounting.

**Table 2:** Main physico-chemical properties of the selected soils.

Soil ID	Depth (cm)	Soil Texture and Classification (USDA)				*ρ_b_* (g/cm^3^)	*OC* (g/kg)	*pH*
		Texture	Sand (%)	Silt (%)	Clay (%)			
SAND	0–20	sand	98	1.5	0.5	1.02	4.5	7.9
SALO	0–20	sandy-loam	57.43	31.95	10.62	1.02	9.5	7.7
SILO	0–20	silty-loam	15.7	72.7	11.6	1.02	26.4	8.4

**Table 3:** Estimated regression coefficients *a* and *b*, and coefficient of determination (*R*^2^) of θ vs. 1/*V_out_* experimental relationships, for SALO, SILO, and SAND soils, in the temperature range 20–30 °C.

Soil Temperature (°C)	SALO *a*, *b*, *R*^2^	SILO *a*, *b*, *R*^2^	SAND *a*, *b*, *R*^2^
20	0.1028, −0.0221, 0.95	0.0844,−0.0102, 0.93	0.0106, −0.0261, 0.95
21	0.0979, −0.0174, 0.94	0.0894, −0.0144, 0.94	0.0886, −0.0044, 0.90
22	0.0182, −0.0147, 0.95	0.0782, −0.0010, 0.95	0.0812, −0.0045, 0.90
23	0.0924, −0.0108, 0.95	0.0862, −0.0117, 0.95	0.0893, −0.0110, 0.93
24	0.0892, −0.0091, 0.92	0.0788, −0.0061, 0.93	0.0809, −0.0195, 0.90
25	0.0733, 0.0049, 0.94	0.074, −0.0067, 0.96	0.0672, −0.0004, 0.94
26	0.0913, −0.0580, 0.95	0.0670, 0.0160, 0.93	0.0821, −0.0129, 0.92
27	0.0990, −0.0180, 0.95	0.0835, −0.0145, 0.91	0.0787, −0.0224, 0.95
28	0.0826, −0.0130, 0.95	0.0766, 0.0920, 0.93	0.0790, −0.0253, 0.94
29	0.0851, −0.0135, 0.94	0.0683, 0.0050, 0.94	0.0687, −0.0150, 0.92
30	0.0699, −0.0035, 0.95	0.0561, 0.0238, 0.95	0.0544, 0.0016, 0.95

**Table 4:** Statistical indicators, mean bias error (*MBE*), mean absolute percentage error (*MAE*), and model efficiency (*EF*) computed by comparing (a) values measured via the thermo-gravimetric method versus θ-MoCAP60, and (b) θ-TDR versus θ-MoCAP60-measurements, for the three selected soils across all soil temperatures.

**(a) Soil Temperature (°C)**	**SALO** ***MBE*, *MAE*, *EF***	**SILO** ***MBE*, *MAE*, *EF***	**SAND** ***MBE*, *MAE*, *EF***
20	−7.21 × 10^−5^, 2.81, 0.93	6.45 × 10^−4^, 3.73, 0.91	4.50 × 10^−3^, 2.74, 0.94
21	−2.06 × 10^−2^, 2.89, 0.92	7.19 × 10^−4^, 2.58, 0.94	−1.67 × 10^−2^, 2.90, 0.90
22	−5.28 × 10^−3^, 2.54, 0.94	−1.90 × 10^−2^, 2.78, 0.93	−1.65 × 10^−2^, 2.77, 0.90
23	3.19 × 10^−5^, 2.07, 0.95	6.61 × 10^−4^, 2.25, 0.95	−4.57 × 10^−3^, 2.11, 0.94
24	−1.91 × 10^−3^, 3.21, 0.92	−7.92 × 10^−3^, 3.19, 0.92	−4.57 × 10^−3^, 2.84, 0.91
25	−1.09 × 10^−2^, 2.46, 0.94	6.28 × 10^−4^, 2.84, 0.94	−4.69 × 10^−3^, 2.57, 0.92
26	−1.78 × 10^−4^, 2.45, 0.95	8.16 × 10^−4^, 3.06, 0.92	−4.43 × 10^−3^, 2.43, 0.94
27	7.07 × 10^−5^, 3.13, 0.92	8.38 × 10^−4^, 3.34, 0.91	1.44 × 10^−2^, 2.24, 0.95
28	1.56 × 10^−4^, 2.51, 0.95	−2.18 × 10^−2^, 3.71, 0.90	−4.52 × 10^−3^, 2.69, 0.94
29	−4.08 × 10^−5^, 2.95, 0.94	−1.43 × 10^−2^, 2.52, 0.93	−4.57 × 10^−3^, 2.60, 0.93
30	−2.40 × 10^−5^, 2.96, 0.93	6.17 × 10^−4^, 2.69, 0.93	−7.99 × 10^−3^, 2.55, 0.93
Overall *	**−3.87 × 10^−3^, 2.72, 0.94**	**−1.16 × 10^−2^, 2.98, 0.93**	**−5.86 × 10^−3^, 2.58, 0.93**
**(b) Soil Temperature (°C)**	**SALO** ***MBE*, *MAE*, *EF***	**SILO** ***MBE*, *MAE*, *EF***	**SAND** ***MBE*, *MAE*, *EF***
20	8.96 × 10^−3^; 3.04; 0.94	−6.45 × 10^−3^; 3.64; 0.88	−1.47 × 10^−2^; 1.54; 0.97
21	2.76 × 10^−2^; 3.54; 0.91	−6.52 × 10^−3^; 2.50; 0.95	−3.57 × 10^−3^; 2.16; 0.95
22	1.37 × 10^−2^; 2.85; 0.93	1.15 × 10^−2^; 2.13; 0.96	−3.76 × 10^−3^; 2.65; 0.92
23	8.87 × 10^−2^; 2.41; 0.95	−6.46 × 10^−3^; 2.17; 0.96	−1.46 × 10^−2^; 2.22; 0.94
24	1.06 × 10^−2^; 2.97; 0.93	1.34 × 10^−3^; 2.90; 0.94	−1.46 × 10^−2^; 2.61; 0.90
25	1.88 × 10^−2^; 3.09; 0.92	−6.44 × 10^−3^; 2.75; 0.94	−1.45 × 10^−2^; 2.52; 0.92
26	9.06 × 10^−3^; 2.32; 0.95	−6.61 × 10^−3^; 1.57; 0.96	−1.47 × 10^−2^; 2.78; 0.92
27	8.83 × 10^−3^; 3.26; 0.91	−6.63 × 10^−3^; 2.84; 0.92	−3.19 × 10^−2^; 3.19; 0.90
28	8.75 × 10^−3^; 2.54; 0.95	1.39 × 10^−2^; 3.73; 0.90	−1.47 × 10^−2^; 1.77; 0.94
29	8.93 × 10^−3^; 2.85; 0.94	7.11 × 10^−3^; 2.30; 0.96	−1.46 × 10^−2^; 2.25; 0.91
30	8.92 × 10^−3^; 3.26; 0.92	−6.43 × 10^−3^; 1.38; 0.97	−1.15 × 10^−2^; 1.90; 0.96
Overall *	**1.21 × 10^−2^; 2.92; 0.93**	**−1.06 × 10^−3^; 2.54; 0.94**	**−1.39 × 10^−2^; 2.33; 0.93**

* i.e., calculated across the temperature domain.

**Figure A1:**
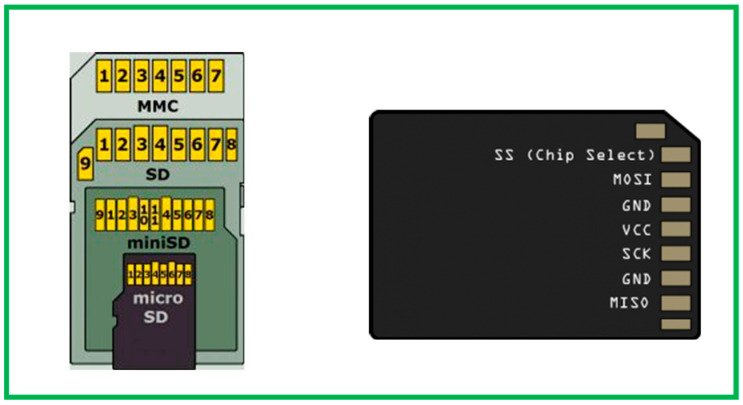
Example of miniSD and microSD pins, and size factor.

**Figure A2:**
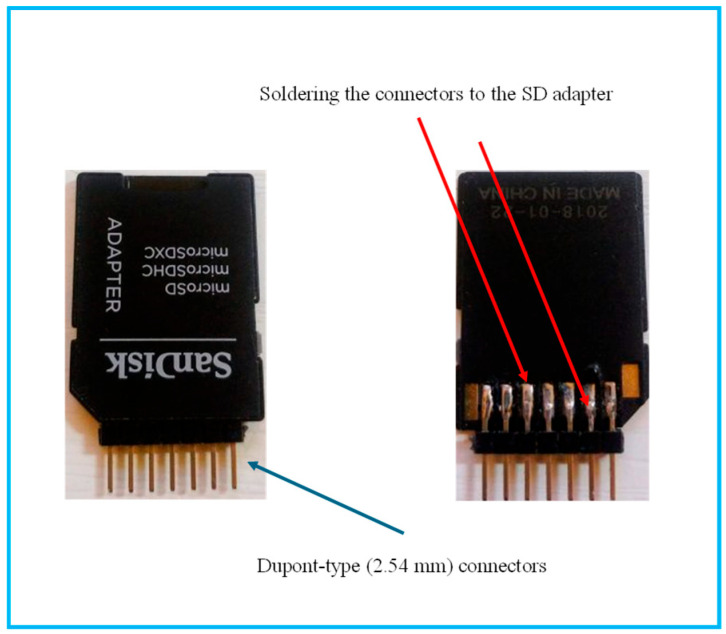
Example microSD-to-SD adapter with soldered pins.

**Figure A3:**
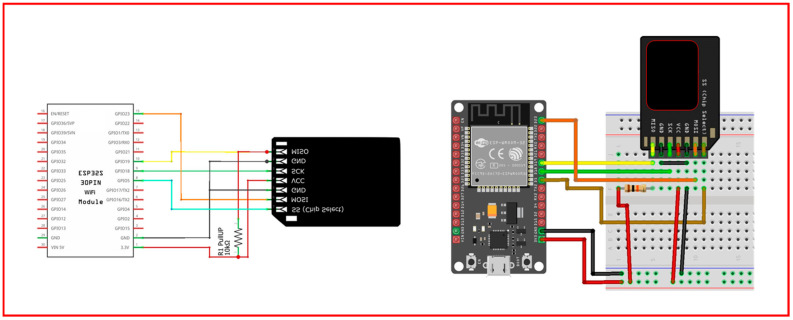
Interfacing microSD adapter to ESP32 microcontroller.

**Figure A4:**
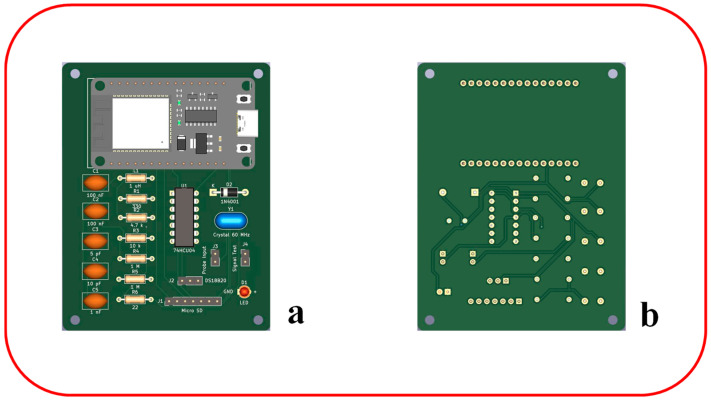
PCB assembled with all the electronic components: (**a**) front panel, (**b**) back panel.

## Data Availability

The original contributions presented in the study are included in the article/Supplementary Materials. Further inquiries can be directed to the corresponding author.

## Supplementary Materials

The following supporting information can be downloaded at: https://www.mdpi.com/article/10.3390/s26051494/s1.

## Appendix A. Construction of a Low-Cost External microSD Module

The SD card supports both its native host interface and an SPI (Serial Peripheral Interface) mode for communication with master devices. The native interface uses four data lines and requires a dedicated SD-host controller within the microcontroller, which often involves licensing constraints. For this reason, being a widely adopted protocol and available on most low-cost microcontrollers, SPI represents the preferred interface in embedded, low-cost applications.

This section outlines a simple procedure for building a low-cost DIY microSD adapter, that can be connected to ESP32 boards.

### Appendix A.1. Pinout

While SD cards come in different form factors, the fundamental pinout remains the same, as reported in Figure A1:

**Table A1:** Pinout configuration of the SD card interface, showing the corresponding functions in native SD mode and SPI mode.

Pin Number	Pin Name	SD Mode Function	SPI Mode Function
1	DAT2/X	Data Line 2	Not used
2	DAT3/CS	Data Line 3	Chip Select
3	CMD/DI	Command/Response Line	Data Input
4	VDD	+3.3 V Power Supply	+3.3 V Power Supply
5	CLK/SCLK	Clock	Serial Clock
6	VSS	Ground	Ground
7	DAT0/D0	Data Line 0	Data Output
8	DAT1/X	Data Line 1	Not used

### Appendix A.2. Wiring Considerations

Since the ESP32 operates natively at 3.3 V (the operating voltage range of the SD family spans from 2.7 V to 3.6 V), interfacing an SD card is significantly simpler compared to a 5 V Arduino board, which would require level shifting. For the hardware connection, a microSD–to–SD adapter was used as the physical interface, resulting in the configuration illustrated in Figure A2 below. All the pins shown in the figure must be soldered to 2.54 mm-pitch Dupont-type connectors.

Once the adapter has been assembled, its connection to an ESP32 board must follow the wiring diagram shown in Figure A3.

## Appendix B. Supplementary Information

This section provides an overview of how the Supplementary Material accompanying this work is organized, which enables the construction of the MoCAP60 sensor. All essential resources required for constructing the device, such as PCB manufacturing files, firmware, and 3D printing models are organized as follows:

**1. Board Fabrication Files folder**:

(a) The Gerber files (MoCAP60.kicad_pcb_gerber.zip);
(b) The Centroid file (MoCAP60.kicad_pcb_positions);
(c) The BOM (MoCAP60.kicad_pcb_bom).

These files are fundamental for PCB implementation in specialized electronics manufacturing services. Note that the PCB is a two-layer version measuring 65 mm × 85 mm.

**2. MoCAP60 Firmware folder**:

(a) Source code developed in Arduino IDE used to program the proposed monitoring system (file name: MoCAP60.ino).

**3. 3D Printing Materials folder**:

MoCAP60 Folder: stl files for quickly assembling MoCAP60 sensor.

-User guide for MoCAP60 sensor.

The 3D-printable models were designed using Fusion 360 software (vers., 36.2606.1.0, https://www.autodesk.com), the user guide contains all the information needed to correctly assemble the MoCAP60 sensor.

Using a specialized PCB fabrication service is recommended. To proceed, the Gerber files must be provided, and if component assembly is required, the Bill of Materials (BoM) must be provided as well. Figure A4 shows the MoCAP60-PCB with all components already mounted.

### Appendix B.1. Details on Technical Specifications and Operating Characteristics of the MoCAP60 Sensor

For the sake of completeness, Table A2 shows the main technological characteristics of the MoCAP60 sensor.

**Table A2:** Main technical specifications and operating characteristics of the MoCAP60 sensor.

Parameter	MoCAP60
Operating temperature range (°C)	20–30
Measurement range (VWC: θ)	0–40
Operating frequency (MHz)	60
PCB structure	2-layer PCB
PCB dimensions (mm)	65 × 85
Power supply	3.7 V, 1000 mAh lithium-ion battery
Current consumption	~20 mA at 3.7 V
Transmission technology	USB-C cable
Estimated cost for PCB (USD/unit)	~0.50–1.0
