# Supplementary material for: Development of a 3D-Printed Capacitive Sensor for Soil Water Content Estimation Using Nickel-Based Conductive Paint
Source: Sensors (Basel). 2026 Feb 27;26(5):1494. doi: 10.3390/s26051494 (PMC12987182; doi:10.3390/s26051494)
Supplement: Supplementary file 1 [file sensors-26-01494-s001.zip › User guide for MoCAP60 sensor.pdf]

Users Guide for Assembling the MoCAP60 Sensor

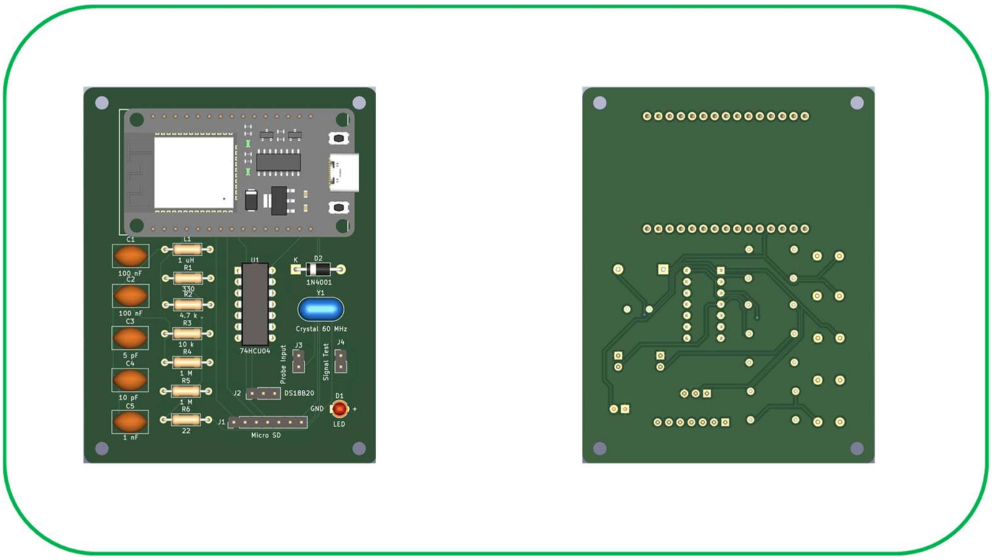

by Prof. Eng. Alessandro COMEGNA

Department of Agricultural, Forest, Food and Environmental Sciences (DAFE),

University of Basilicata, Potenza, Italy

Version 1.0 (2026)

This brief guide provides details for assembling the MoCAP60 capacitive sensor.

It also describes the 3D-printed components and PCB files required for building the complete device.

1. Source Code for the MoCAP60 Sensor

The MoCAP60 sensor operates using an ESP32 mini (30 Pins) microcontroller programmed in C++ within the Arduino IDE environment.

The firmware controls the capacitive module, temperature readings, and data storage on microSD.

file name: *MoCAP60.ino* (see folder in supplementary material: **MoCAP60 Firmware**).

Below is the full and complete listing of the firmware necessary for the operation of the MoCAP60 device.

```
////////////////CODE General Information/////////
// MoCAP60 sensor (Moisture Capacitive sensor; works at 60 MHz)
//
// This firmware have to be uploaded on the ESP32 mini to utilize the MoCAP60 sensor
//
// Sensors:
// - DS18B20 Temperature Sensor (OneWire protocol)
// - Capacitive Soil Moisture Sensor (analog reading) - MoCAP60
//
// Features:
// - Reads temperature in Celsius
// - Measures soil water content using capacitive sensing at 60 MHz
```

```
// - Calculates volumetric water content (TETA) using calibration formula
// - Stores data to SD card in CSV format
// - Supports multiple readings with averaging for stability
//
// Data Format:
// readingID, SWC_raw, SWC_voltage, SWC_TETA, temperature
//
// Pin Configuration:
// - SD Card CS: Pin 5
// - DS18B20: Pin 21
// - Soil Moisture Sensor: Pin 34 (ADC) - MoCAP60
//
// Required Libraries:
// - FS.h
// - SD.h
// - SPI.h
// - OneWire.h
// - DallasTemperature.h
// Author: Alessandro Comegna (email: alessandro.comegna@unibas.it)
// version 1.0 (January 2026)
///////////////////////////////////////////////////////////////////////////////////////////
/////////////////////////////
```

```
// Libraries for SD card
#include "FS.h"
#include "SD.h"
#include <SPI.h>

//DS18B20 libraries
#include <OneWire.h>
#include <DallasTemperature.h>

// Define CS pin for the SD card module
#define SD_CS 5

int readingID = 0;
////////////////////////SWC: Soil Water Content Capacitive Module//////////////////////////////
const int SWCPin = 34; // 12 bits ADC pin 4 senses the voltage level of the MoistSensor1 (values 0 - 4095)
int SWC = 0; // Variable to store the Moist level
float  SWC_volt=0.0 ;
float SWC_TETA = 0.0; // Variable to store the Moist level
///////////////////////////////////////////////////////////////////////////////////
//
```

```
String dataMessage;

///////////////////////Temperature

Module///////////////////////////////////////

#define ONE_WIRE_BUS 21 // modificare perchè già preso da sim800

// Setup a oneWire instance to communicate with a OneWire device

OneWire oneWire(ONE_WIRE_BUS);

// Pass our oneWire reference to Dallas Temperature sensor

DallasTemperature sensors(&oneWire);

float temperature;

////////////////////////////////////////////////////////////////////////////////


void setup() {


    sensors.begin();

  Serial.begin(115200);


 ///////////////////////////   SWC   ///////////////////////////

   pinMode(SWCPin, INPUT);             // Initializes the sensor pin (4) for measuring the

MoistureLevelValue1

 ///////////////////////////////////////////////////////////////////////////
```

```
// Initialize SD card
Serial.print("Card Initialization: ");
SD.begin(SD_CS);
if(!SD.begin(SD_CS)) {
  Serial.println("Card Mount Failed");
  return;
}
uint8_t cardType = SD.cardType();
if(cardType == CARD_NONE) {
  Serial.println("No SD card attached");
  return;
}
Serial.println("Initializing SD card...");
if (!SD.begin(SD_CS)) {
  Serial.println("ERROR - SD card initialization failed!");
  return;    // init failed
}

// If the data.txt file doesn't exist
// Create a file on the SD card and write the data labels
File file = SD.open("/data.txt");
if(!file) {
```

```
    Serial.println("File doens't exist");
    Serial.println("Creating file...");
    writeFile(SD, "/data.txt", "Temperature \r\n");
  }
  else {
    Serial.println("File already exists");
  }
  file.close();

    // Increment readingID on every new reading
//  readingID++;
  }

void loop() {
  readingID++;
                                             //////////////////////////////////////Temperature
Readings//////////////////////////////
  sensors.requestTemperatures();
  temperature = sensors.getTempCByIndex(0); // Temperature in Celsius
  delay(200);
 //////////////////////////////////////////////////END                   TEMPERATURE
Readings/////////////
```

```
/////////////////////////////////////////////////////////////////////////////

  analogRead(SWCPin); /* Insert a pause between two analogic measurements */

///////////////////Resistive                                                                    sensor
module//////////////////////////////////////////////////


///////////////////////////////////////SWC
Readings/////////////////////////////////////
SWC= 0;

 for (int m = 1; m < 50 ; m++)                    // take 50 consecutive measurements
   {
     SWC = SWC + analogRead(SWCPin) ;    // Read data from analog pin 34 and add it to
MoistLevel1 variable
     delay (1000);
   }

  SWC = SWC / 50;                    // Determine the average of 5 measurements
  SWC_volt = SWC * (3.3 / 4096.0);     //ADC convert 12 bit at 3.3 volts input
 // SWC_volt = SWC * (5.0 / 4096.0);     //ADC convert 12 bit at 5.0 volts input
```

```
SWC_TETA=0.0672*(1/SWC_volt)-0.0004;   // calibration function

/////////////////////////////////////End                                   SWC

Readings/////////////////////////////////////


   /////////////////////OUTPUT INITIALIZATION//////////////////////////


///////////////SWC SENSOR OUTPUT///////////////


 Serial.print("\n");

 Serial.print(" Avg. value = ");

 Serial.print(SWC);

 Serial.print("\n");

 Serial.print(" volts = ");

 Serial.print(SWC_volt);

 Serial.print("\n");

 Serial.print(" moist = ");

 Serial.print(SWC_TETA);

 Serial.print("\n");

          ////////////////////////TEMPERATURE          (DS18B20)          SENSOR

OUTPUT//////////////////////

 Serial.print("Temperature: ");

 Serial.print(temperature);
```

```
  Serial.println(" °C");

///////////////////SD CARD OUTPUT MODULE/////////////////////////////
// Write the sensor readings (i.e. datamessage) on the SD card
  dataMessage = String(readingID) + "," + String(SWC)+ "," + String(SWC_volt) +"," + String(SWC_TETA) +"," + String(temperature) + "\r\n"; //inserire qui string(now.year(), DEC)+ "," + string(now.month(), DEC)+ "," + string(now.day(), DEC)+ string(now.minute(), DEC) string(now.hour(), DEC)+
  Serial.print("Save data: ");
  Serial.println(dataMessage);
  appendFile(SD, "/data.txt", dataMessage.c_str());
//////////////////End SD Card///////////////////////////////////
}                                   ////END LOOP

// Write to the SD card (DON'T MODIFY THIS FUNCTION)
void writeFile(fs::FS &fs, const char * path, const char * message) {
  Serial.printf("Writing file: %s\n", path);

  File file = fs.open(path, FILE_WRITE);
  if(!file) {
    Serial.println("Failed to open file for writing");
    return;
```

```
 }
 if(file.print(message)) {
  Serial.println("File written");
 } else {
  Serial.println("Write failed");
 }
 file.close();
}

// Append data to the SD card (DON'T MODIFY THIS FUNCTION)
void appendFile(fs::FS &fs, const char * path, const char * message) {
 Serial.printf("Appending to file: %s\n", path);

 File file = fs.open(path, FILE_APPEND);
 if(!file) {
  Serial.println("Failed to open file for appending");
  return;
 }
 if(file.print(message)) {
  Serial.println("Message appended");
 } else {
  Serial.println("Append failed");
```

```
 }
 file.close();
}
```

2. MoCAP60 Assembling

The MoCAP60 sensor consists of:

- A 3D-printed PLA sensor body.

- Two PLA guides coated with a nickel-based conductive paint, which serve as the capacitive electrodes.

- A custom PCB hosting the measurement circuit.

- A microSD adapter for data logging (see appendix A for building a low-cost DIY microSD adapter).

- Electrical wiring and connectors.

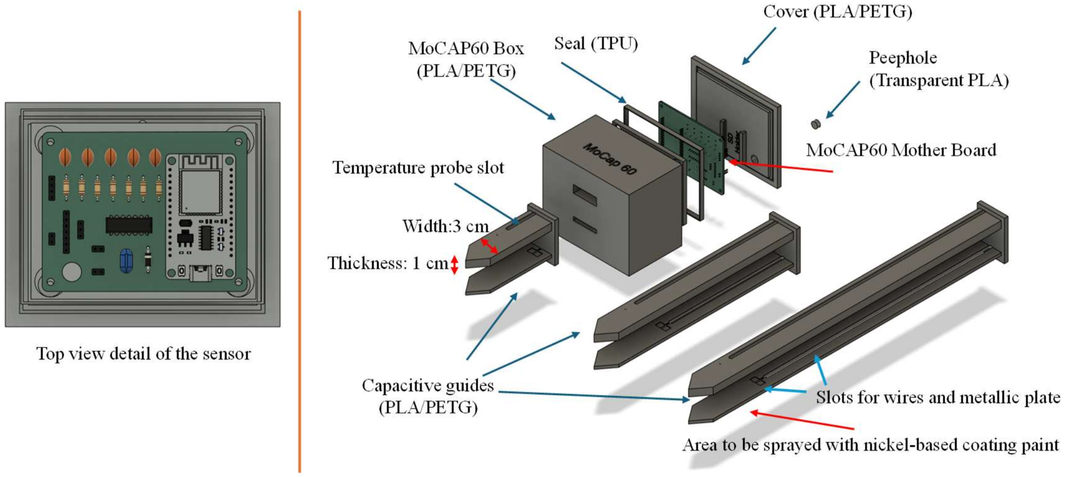

3. 3D-Printed Components

All 3D files, listed below, are provided in the **3D_MoCAP60 folder**.

- Bottom Box (PLA).

✓ Bottom End (PLA).

- ✓ Cap (TPU).
- ✓ Cover (PLA).
- ✓ Peephole (PLA Tranlucent).
- ✓ PROBE (PLA) (5 cm).
- ✓ PROBE (PLA) (15 cm).
- ✓ PROBE (PLA) (30 cm).
- ✓ Seal (TPU).
- ✓ Cover for 15 cm waveguides (PLA x2).
- ✓ Cover for 30 cm waveguides (PLA x2).

4. Electrodes and Wiring

The sensor uses two rectangular PLA plates, positioned on opposite sides of the 3D-printed body.

5. PCB Board

The PCB_MoCAP60 folder contains Gerber files, BoM, and assembly drawing.

MoCAP60.kicad_pcb_bom.

MoCAP60.kicad_pcb_gerber (zip folder).

MoCAP60.kicad_pcb_netlist.

MoCAP60.kicad_pcb_positions.

6. Assembly Procedure

1. Print all 3D components.
2. Assemble the capacitive electrodes.
3. Install the internal wiring.
4. Mount the PCB inside the 3D body.

5. Connect the microSD adapter.

6. Close the sensor using the printed top cover.

7. Upload the firmware to the ESP32.

8. Run the calibration procedure.

9. Perform the validation experiment.

Appendix – Fabrication of the Capacitive Guides

This appendix provides detailed instructions for manufacturing the capacitive guides used in the MoCAP60 sensor. These guides act as the electrodes responsible for generating the capacitive field and enabling soil moisture estimation.

A1. Materials

The following materials are required:

-PLA guides (3D-printed using the files:

- ✓ PROBE (PLA) (5 cm)

or

- ✓ PROBE (PLA) (15 cm).

or

- ✓ PROBE (PLA) (30 cm).

-Nickel-based conductive paint (spray application)

- ✓ Nitrile gloves and protective mask
- ✓ Electrical wires (24–28 AWG)
- ✓ Cyanoacrylate adhesive or epoxy resin

A2. Surface Preparation

Before applying the conductive coating, the PLA surfaces should be cleaned to ensure proper adhesion:

1. Remove any printing residues from the PLA guides.
2. Clean the surface with a dry cloth to remove plastic dust.
3. Cover the probe parts that should not be coated with paint.

A3. Wiring the Electrodes

1. Insert the AWG wires into the dedicated sockets.
2. Solder one end of the wire to the metallic plate.
3. Apply a small amount of conductive epoxy or solder paste (if applicable) to the designated contact point (i.e., the socket for the metallic plate).

The resulting structure forms the two plates of the capacitive sensor.

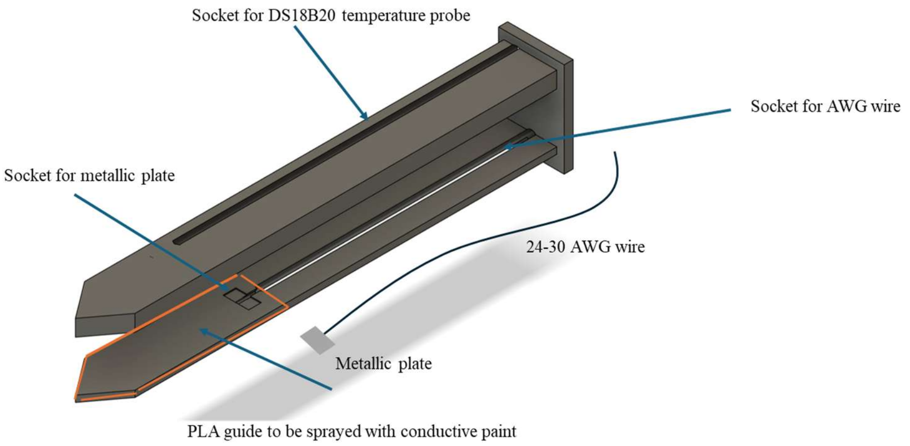

A4. Application of Nickel-Based Conductive Paint

Once the wiring has been completed, proceed with the conductive paint, which is essential to transform the PLA supports into functional electrodes.

1. Shake the nickel-based paint spray can for at least 1–2 minutes.
1. Place the PLA guides on a clean, protected surface.
2. Apply **two or three uniform layers** of conductive paint, keeping the spray at a distance of 10–15 cm to avoid drips.
3. Allow each layer to dry for approximately 10–15 minutes.
4. Verify that the coating is homogeneous and covers the entire electrode surface.

5. In the case of using the 15 cm or 30 cm waveguides, cover the inside of the capacitive guides with the designated cover (see files *cover for 15 cm waveguides.stl*; *cover for 30 cm waveguides.stl*) and glue each cover to the waveguides.

A5. Integration into the Sensor Body

1. Insert the capacitive guides into the dedicated slots of the 3D-printed sensor body.
2. Fix the guides using adhesive if necessary the PLA support (Bottom Box PLA file).
3. Route the wires through the internal channels and connect them to the PCB following the pin assignment.

A6. Final Inspection

1. Check electrical continuity using a multimeter.
2. Verify that the two electrodes are positioned parallel to each other. This check should be performed carefully also when inserting the sensor probes into the soil.
3. Ensure that the conductive paint layer is intact and free of scratches.

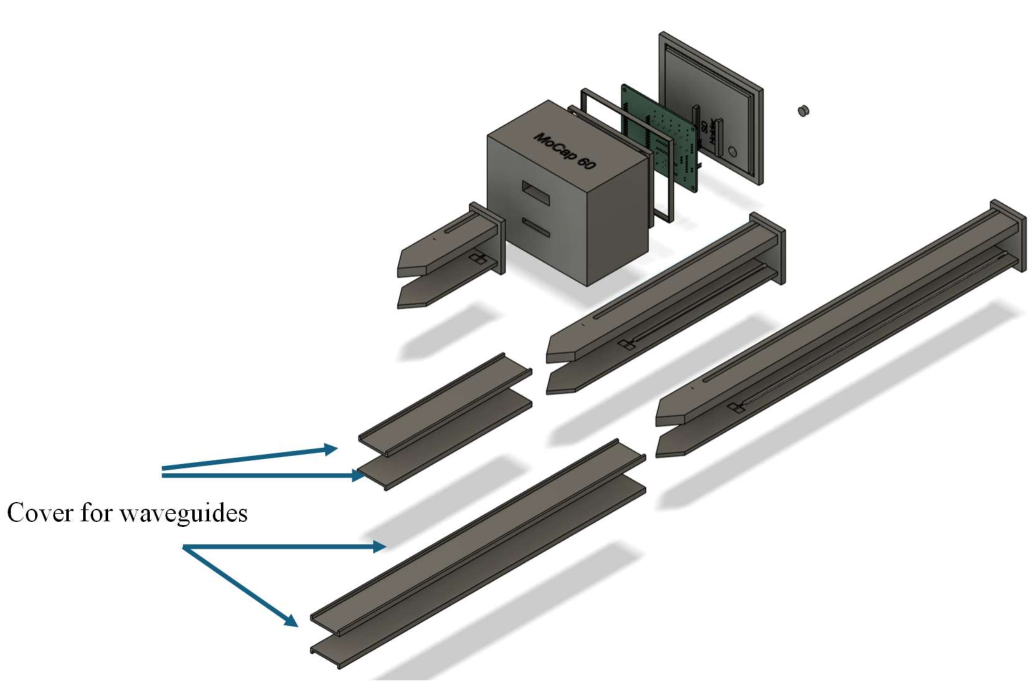

Guide for MoCAP60 sensor
