# Supplementary figures and images for: Development of a 3D-Printed Capacitive Sensor for Soil Water Content Estimation Using Nickel-Based Conductive Paint
Source: Sensors (Basel). 2026 Feb 27;26(5):1494. doi: 10.3390/s26051494 (PMC12987182; doi:10.3390/s26051494)

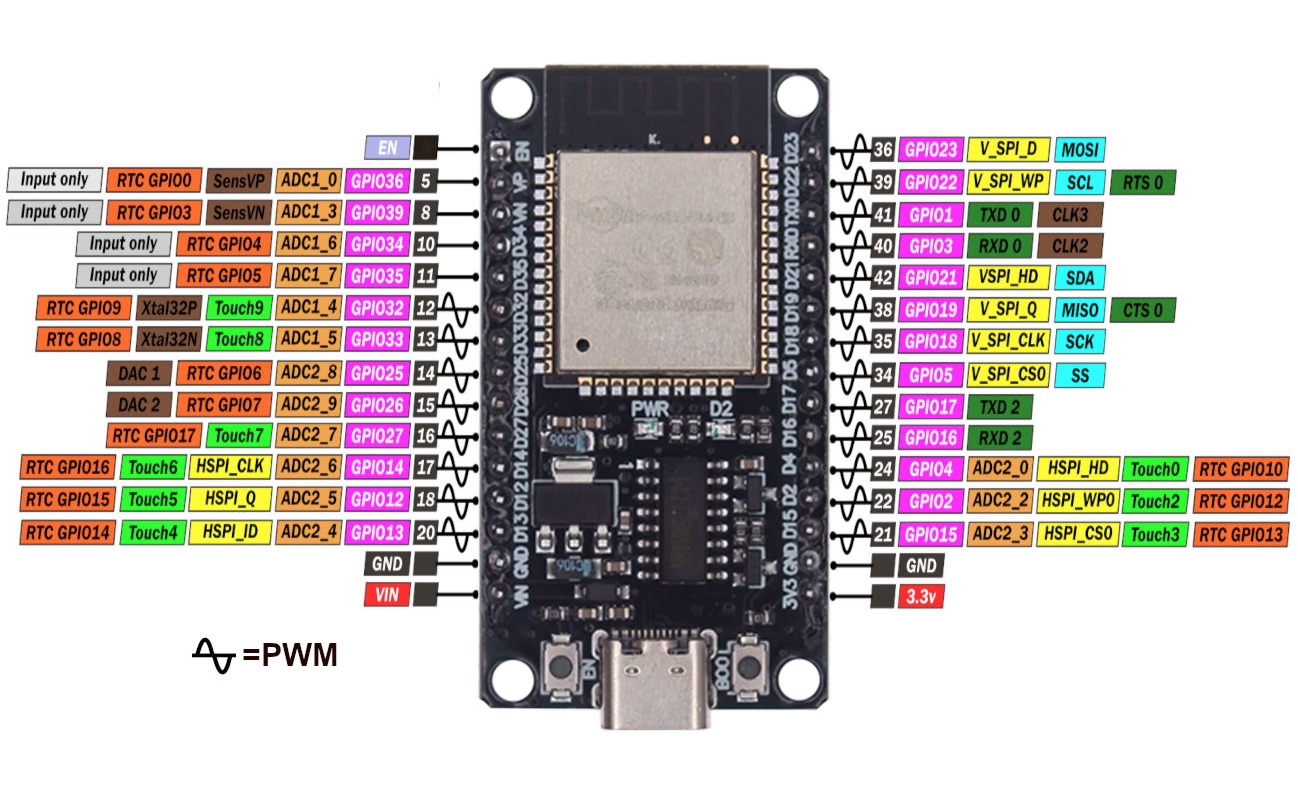

Supplement: Supplementary file 1 [file sensors-26-01494-s001.zip › esp32 usbc pinout.jpg]

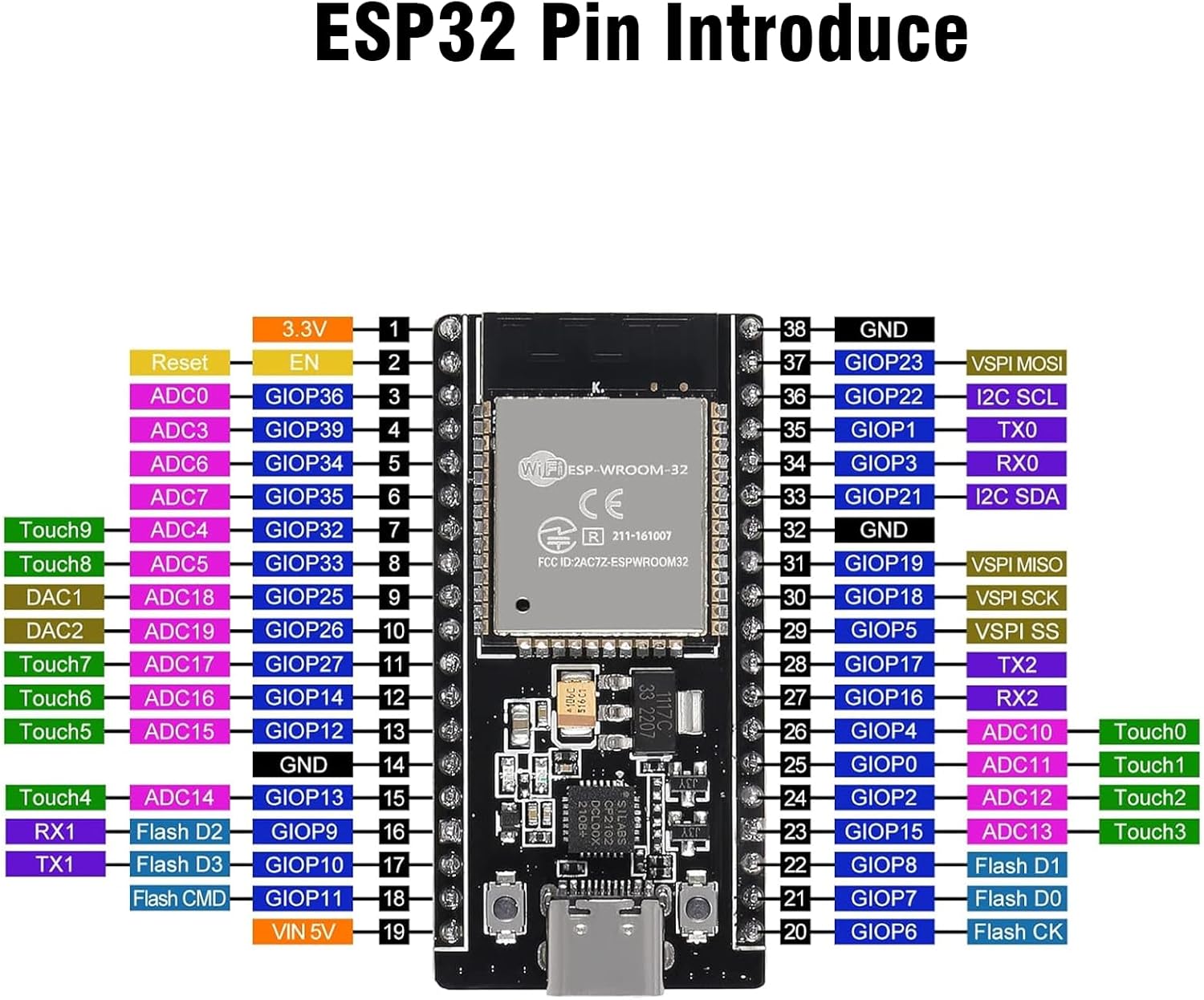

Supplement: Supplementary file 1 [file sensors-26-01494-s001.zip › PINOUTESP32UBSC.jpg]
